# Responses of growth and photosynthesis to alkaline stress in three willow species

**DOI:** 10.1038/s41598-024-65004-5

**Published:** 2024-06-25

**Authors:** Shenqi Qiao, Changming Ma, Hongjiao Li, Yu Zhang, Minghui Zhang, Wenhao Zhao, Bingxiang Liu

**Affiliations:** 1 Department of Forest Cultivation, College of Forest, Hebei Agricultural University, Baoding, 071000 China; 2 College of Life Science, Hengshui, Hebei, China; 3 Hebei Urban Forest Health Technology Innovation Center, No. 2596 Lekai South Street, Lianchi District, Baoding City, 071000 Hebei Province China

**Keywords:** Three willow cuttings, Alkaline stress, Root and leaf dry weight, Root and leaf water content, Chlorophyll content, Photosynthesis, Chlorophyll fluorescence kinetic parameters, Forestry, Plant sciences, Light responses, Photosynthesis, Plant physiology, Plant stress responses

## Abstract

Investigating differences in resistance to alkaline stress among three willow species can provide a theoretical basis for planting willow in saline soils. Therefore we tested three willow species (*Salix matsudana*, *Salix gordejevii* and *Salix linearistipularis*), already known for their high stress tolerance, to alkaline stress environment at different pH values under hydroponics. Root and leaf dry weight, root water content, leaf water content, chlorophyll content, photosynthesis and chlorophyll fluorescence of three willow cuttings were monitored six times over 15 days under alkaline stress. With the increase in alkaline stress, the water retention capacity of leaves of the three species of willow cuttings was as follows: *S. matsudana* > *S. gordejevii* > *S. linearistipularis* and the water retention capacity of the root system was as follows: *S. gordejevii* > *S. linearistipularis* > *S. matsudana*. The chlorophyll content was significantly reduced, damage symptoms were apparent. The net photosynthetic rate (Pn), rate of transpiration (E), and stomatal conductance (Gs) of the leaves showed a general trend of decreasing, and the intercellular CO_2_ concentration (Ci) of *S. matsudana* and *S. gordejevii* first declined and then tended to level off, while the intercellular CO_2_ concentration of *S. linearistipularis* first declined and then increased. The quantum yield and energy allocation ratio of the leaf photosystem II (PSII) reaction centre changed significantly (φPo, Ψo and φEo were obviously suppressed and φDo was promoted). The photosystem II (PSII) reaction centre quantum performance index and driving force showed a clear downwards trend. Based on the results it can be concluded that alkaline stress tolerance of three willow was as follows: *S. matsudana* > *S. gordejevii* > *S. linearistipularis*. However, since the experiment was done on young seedlings, further study at saplings stage is required to revalidate the results.

## Introduction

Soil salinisation is one of humanity's most severe environmental problems and a pivotal constraint to agroforestry development^1^. Globally, the area of saline soil is approximately 0.64 billion hm^2^ and is growing at a rate of 1.5 × 10^6^ per year^2^. Saline soils in China are mainly distributed on the eastern coast and in the northwestern arid areas^3^, the land use efficiency is low. Therefore, screening alkaline-tolerant plants by experimentally simulating different saline environments significantly improves saline land use efficiency. Most scholars working on screening and evaluating plant alkaline tolerance have focused on growth and development status, microstructure, photosynthetic pigments, photosystems, ion uptake and transport characteristics, and fluorescence parameters^4^. Current studies on the effects of alkaline stress on plants have focused on cash crops such as *Hordeum bogdanii*, *Oryza sativa* L. and *Beta vulgaris* L.^5–7^, with fewer studies on forest trees.

Willow (*Salix babylonica*) is a woody plant of the Salicaceae family distributed in the north temperate zone^8^. Due to its attractive crown shape and green foliage, willow has excellent characteristics, such as fast sprouting, flood resistance, and salinity tolerance. It is widely used in saline-alkaline land reclamation projects. In addition to neutral salts dominated by NaCl, alkaline salts are dominated by Na_2_CO_3_ in the soil^9^. According to Zhao et al.^10^, plants can adapt to alkaline environments by thickening their epidermis and flesh, shrinking their leaves, and altering their tissue structure. The high pH of alkaline stress disrupts plant ion homeostasis, hinders photosynthesis and photosynthetic pigment production, damages biomolecules such as DNA, lipids and proteins, and causes slow growth and even death of plants^11^. Currently, studies on the physiological aspects of stress tolerance mainly focus on the physiological characteristics of pests and diseases, heavy metals, salinity, and water^12^. Little has been reported on the changes in the water content of root and leaf tissues and the photosynthetic system under alkaline stress, while there are few reports on the relationship between the water uptake and utilisation efficiencies of roots and leaves and the photosynthetic system under alkaline stress. This experiment aimed to clarify the changes in the plant tissue water content and photosynthetic system of *S. matsudana*, *S. gordejevii* and *S. linearistipularis* under alkaline stress to clarify the salt tolerance mechanism of the three willow species in regards to tissue water content and photosynthetic system. Comparative analysis of the alkaline stress resistance of the three willow cuttings will provide a theoretical reference for the selection and cultivation of saline and alkaline tree species.

## Materials and methods

### Experimental materials and design

The experimental site was located at the West Campus of Hebei Agricultural University, Baoding City, Hebei Province. The temperature of the climate chamber was set at 28 °C/25 °C (light/dark), light intensity was controlled by an LED cold light source at 1000 μmol m^−2^ s^−1^, photoperiod was 14 h/10 h (light/dark), relative humidity was 60%.

The test materials were collected from the germplasm resource nursery of Jinsha Beach Forestry Farm, Huai'an County, Hebei Province. The branches of three willow species, *S. matsudana*, *S. gordejevii* and *S. linearistipularis*, which were the same in length and strong without pests and diseases, were collected in the early spring before bud break and then stored in a freezer at 0 °C. The hydroponic test was started in the artificial climate chamber of Hebei Agricultural University, Baoding City, Hebei Province. Before the experiment, The middle two-thirds of the selected branches were.

cut into 20 cm-long cuttings. The uppermost bud was 0.5–1 cm from the top of the cuttings. Adequate number of willow cuttings were grown in fresh water and managed regularly. When their growth reached the treatment requirements (average root length of approximately 5 cm), cuttings with uniform growth and root systems were selected to start the alkaline stress treatment experiment.

**Figure 1:**
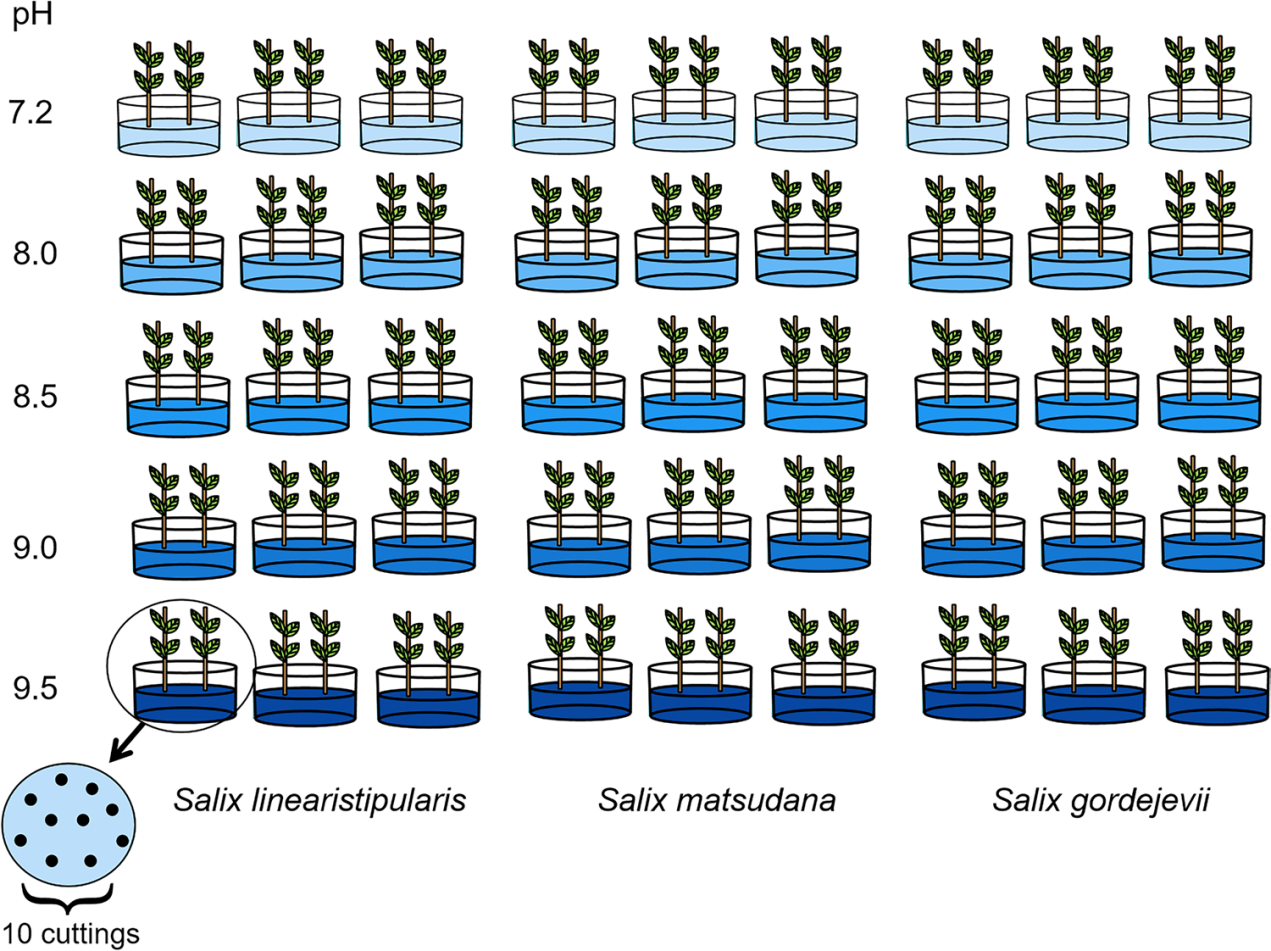
Design of three willows experimental treatment.

The cuttings were placed in 55 cm × 38 cm × 15 cm (L × W × H) plastic boxes for hydroponics with five treatments. Hydroponic solutions with pH values of 8.0, 8.5, 9.0, and 9.5 (mNa_2_CO_3_: mNaHCO_3_ = 1:1) were prepared using 1/2 Hoagland complete nutrient solution as the background solution and 1/2 Hoagland complete nutrient solution as a control (CK). The pH of CK was 7.2. Each treatment was replicated three times, and 20 cuttings were placed in each replication and directly immersed in the solution, approximately half the cuttings' height (Fig. 1). The nutrient solution was changed every 5 days during the growth process. Before the nutrient solution was changed, the cuttings were removed and the roots were rinsed with water to wash away the last residual salt and prevent excessive salt accumulation. We randomly selected three seedlings with even growth vigor on the 1st, 3rd, 5th, 8th, 11th, and 15th days of the stress test for the observation of various growth physiological indexes. The measurement of each index was repeated for 3 times, and the growth status was observed and recorded 1 week after the end of the experiment.

### Measurements of various indicators

The water content of roots and leaves, the chlorophyll content of leaves were measured using the method of Li^13^. The plant samples were all cut off and put in the weighing bottle respectively, then put into the oven to kill at 105 °C for 15 min and then dried at 80–90 °C to constant weight after end of killing. Set the weight of the weighing bottle as W_1_, the weight of the weighing bottle and the plant sample as W_2_, and the weight of the weighing bottle and the dried plant sample as W_3_, water content (%) of the plant tissues can be calculated according to the following formula: total water content of the plant tissues = (W_2_−W_3_)/(W_2_−W_1_) × 100%.

Weigh 0.2 g of leaf blade and soaked in 95% ethanol for 24 h. The chlorophyll extract was poured into a 1 cm aperture cuvette. The absorbance was measured at 665 nm and 649 nm using 95% ethanol as blank. The concentrations of chlorophyll a (1), chlorophyll b (2) and total chlorophyll (3) were then calculated using the following equations.

1 $${\text{C}}_{{\text{a}}} = 12.72{\text{A}}_{665} - 2.59{\text{A}}_{649}$$

2 $${\text{C}}_{{\text{b}}} = 22.88{\text{A}}_{649} - 4.67{\text{A}}_{665}$$

3 $${\text{C}}_{{\text{T}}} = {\text{C}}_{{\text{a}}} + {\text{C}}_{{\text{b}}} = 20.29{\text{A}}_{649} - 8.05{\text{A}}_{665}$$

The chlorophyll fluorescence parameters were measured using the method developed by Ran et al.^14^. The leaves were dark-adapted for 15 min before measurement, and their fast chlorophyll fluorescence induction kinetic curves and related parameters were determined using a Pocket PEA Plant Efficiency Analyser (Hansatech, UK). We performed the following analyses and calculations: initial fluorescence (Fo), maximum fluorescence (Fm) and maximum photochemical efficiency (Fv/Fm) of the leaves after dark adaptation and the performance index (PI_ABS_) based on absorbed light energy.

Photosynthetic parameters were measured using Ran Xin's method^14^. During the stress treatments, three cuttings with uniform growth were selected each time for each treatment. Using a Li-6800 portable photosynthesiser (LI-COR, USA), we measured the stomatal conductance (Gs), intercellular CO_2_ concentration (Ci), transpiration rate (E), and net photosynthetic rate (Pn) in a climatic chamber. We ensured that the leaves had the same size direction of exposure to light and were fully expanded and functional. The measurement conditions were as follows: PAR of 1000 μmol m^−2^ s^−1^, CO_2_ concentration of 400 μmol mol^−1^ in the fixed system, and relative humidity of 60%.

### Data analysis

Plotting was performed using Origin 2021 software, experimental data for root and leaf moisture content of three willow cuttings was analysed using SPSS 18.0 data processing software for statistical analysis. One-way ANOVA and least significant difference (LSD) method was used to check the significance of the difference when the *P*-value was less than 0.05 and 0.01, respectively.

### Germplasm collection license statement

Permission to collect three willow cuttings was obtained prior to the collection of experimental material and is hereby declared.

### Experimental guidelines and license disclaimer

All procedures of the experiment were carried out in accordance with the guidelines, and the whole process of the experiment was approved. The experiment was carried out in accordance with national and regional regulations.

## Results

### Changes in the growth status, root and leaf dry weights of three willow cuttings under alkaline stress

**Figure 2:**
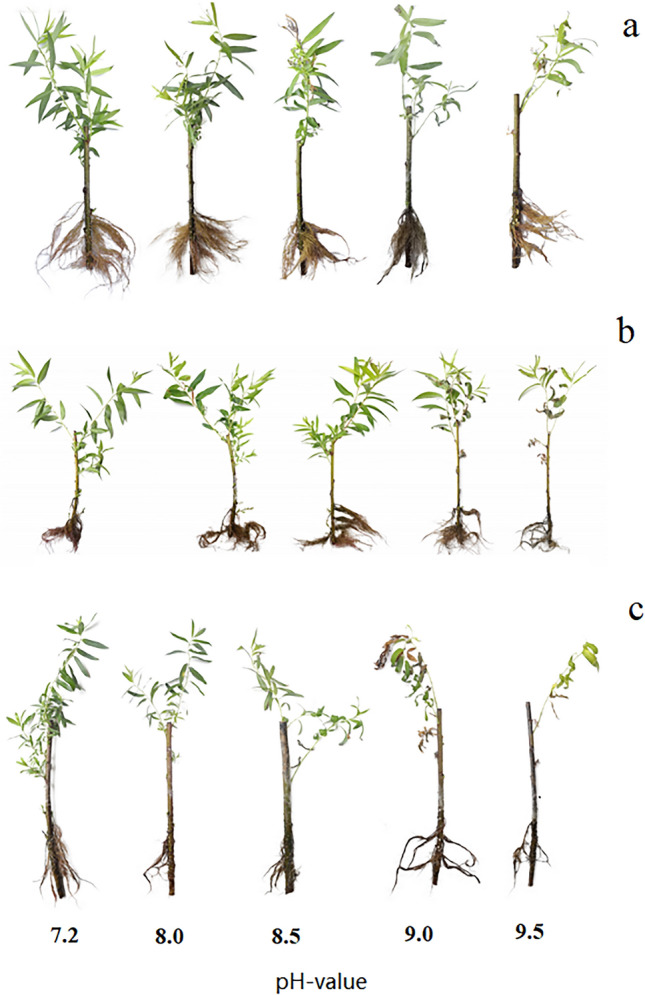
Effects of alkaline stress on the morphology of three willow cuttings (**a**: *S. matsudana*; **b**: *S. gordejevii*; **c**: *S. linearistipularis*). Note: From left to right are the changes of willow cuttings growth with the increase of pH on the 8th day of stress.

Changes in the external morphology of plants can intuitively reflect the strength of their alkaline tolerance. The changes in the growth of the three willow cuttings at different pH values were shown in Fig. 2a, b and c, which showed that the growth damage increased with the increase of pH. The number of damaged leaves gradually increased as the leaf tips, margins or veins of the leaves turned yellow until they wilted and fell off, and the branches dried up with a decrease in the root system, which was severe damage. Comparatively, *S. matsudana* maintained better external morphology at higher pH, followed by *S. gordejevii*, while *S. linearistipularis* suffered most severely under alkaline stress.

The root and leaf dry weights of the three willow cuttings exhibited distinct variations under alkaline stress at different pH levels (Table 1). Specifically, the leaf dry weights of *Salix matsudana* showed significant differences at pH 9.0 compared to the control. Moreover, the leaf dry weight of *Salix gordejevii* and *Salix linearistipularis* significantly differed at pH 8.0 compared to the control. For *Salix matsudana*, the root dry weight only exhibited significant differences from the control at pH 9.0. Conversely, the root dry weights of *Salix gordejevii* and *Salix linearistipularis* significantly differed from the control at pH 8.0 and pH 8.5, respectively. Additionally, across all pH levels, *Salix matsudana* consistently displayed higher root and leaf dry weights, while *Salix linearistipularis* exhibited lower root and leaf dry weights. *Salix gordejevii* showed intermediate values for both root and leaf dry weights, positioning it between the other two willow species.

**Table 1:** Effects of alkali stress on changes in leaf and root dry weight of three willow cuttings. (In statistical terms, the letters a, b, c, and d represent significant differences among the respective data at the *P ≤ 0.05* level).

Targets	Process	Tree species		
	pH	*Salix matsudana*	*Salix gordejevii*	*Salix linearistipularis*
Dry weight of leaf (g)	7.2	0.671 ± 0.049a	0.502 ± 0.177a	0.219 ± 0.039a
	8.0	0.669 ± 0.028a	0.377 ± 0.087b	0.196 ± 0.020b
	8.5	0.626 ± 0.027a	0.322 ± 0.058bc	0.140 ± 0.030c
	9.0	0.577 ± 0.019b	0.282 ± 0.032c	0.126 ± 0.024cd
	9.5	0.493 ± 0.124c	0.179 ± 0.024d	0.114 ± 0.024d
Dry weight of root (g)	7.2	0.384 ± 0.040a	0.185 ± 0.080a	0.070 ± 0.016a
	8.0	0.380 ± 0.027a	0.149 ± 0.049b	0.069 ± 0.011a
	8.5	0.383 ± 0.017a	0.133 ± 0.035b	0.055 ± 0.009b
	9.0	0.364 ± 0.016a	0.125 ± 0.022b	0.053 ± 0.007b
	9.5	0.314 ± 0.087b	0.076 ± 0.020c	0.049 ± 0.007b

### Effects of alkaline stress on the water content of three willow cuttings

**Figure 3:**
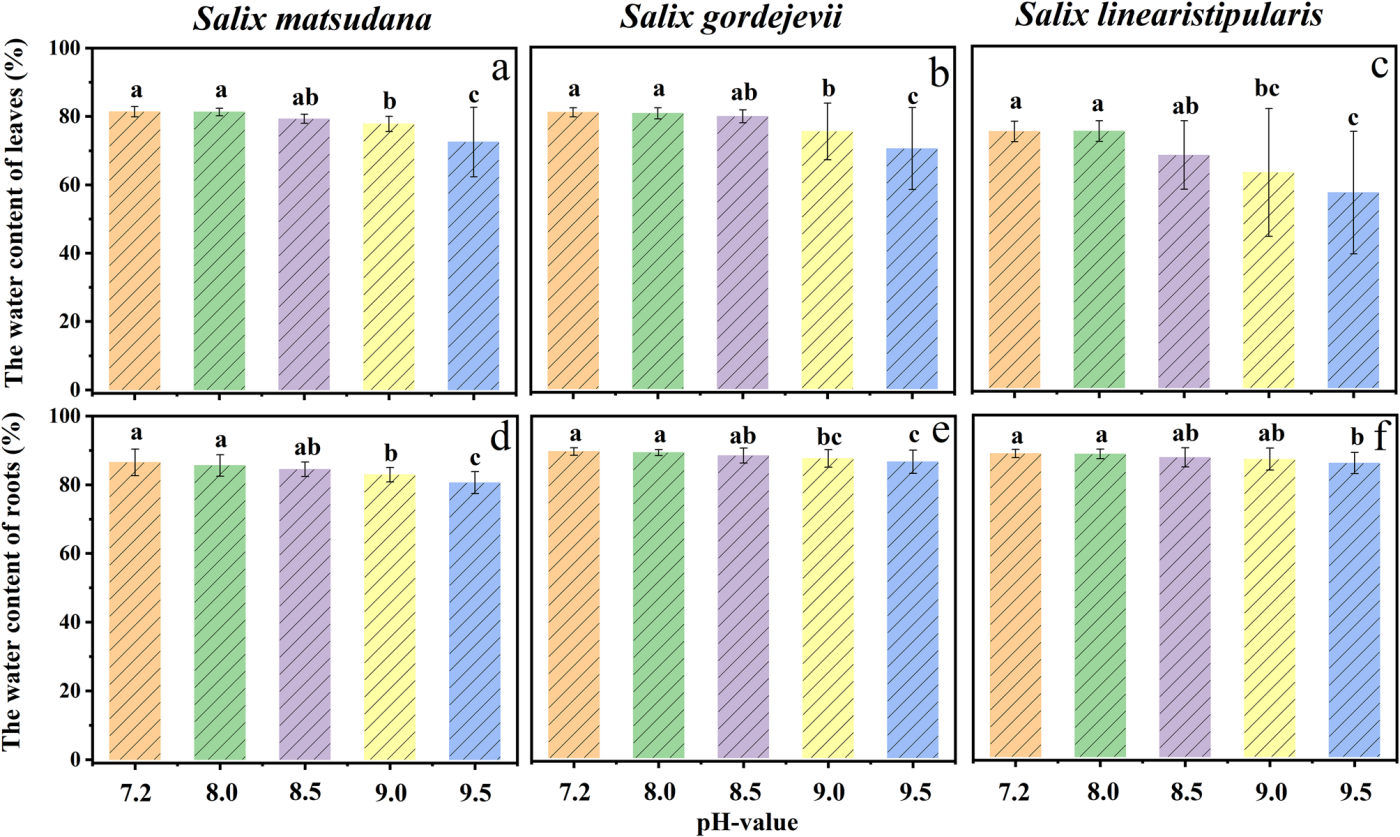
Effects of alkaline stress on the leaf water content (**a**: *S. matsudana* ; **b**: *S. gordejevii*; **c**: *S. linearistipularis*) and root water content (**d**: *S. matsudana*; **e**: *S. gordejevii*; **f**: *S. linearistipularis*) of three willow cuttings.

As in from Fig. 3, the root and leaf water content of the three willow cuttings was affected to different degrees under different concentrations of alkaline stress, and root and leaf water content showed an overall decreasing trend with increasing pH. When the pH of the alkaline solution was 8.0 and 8.5, the leaf water content of the three willow cuttings decreased, but there was no significant difference compared with that of the control group (Fig. 3a, b, c). When the pH of the alkaline solution was 9.0 and 9.5, the leaf water content of the three willow cuttings decreased significantly, with the most significant decrease occurring in *S. linearistipularis* (Fig. 3c). This result indicated that the water retention capacity of *S. linearistipularis* leaves was the worst among the three willow cuttings under high concentrations of alkaline stress.

At alkaline stress pH values of 8.0 and 8.5, there was no significant difference in the root water content of *S. matsudana* and *S. gordejevii* compared with the control (Fig. 3d, e). When the pH was 9.0 and 9.5, the root water content of *S. matsudana* and *S. gordejevii* decreased significantly compared with that of the control. In contrast, the difference in root water content of *S. linearistipularis* was only significant at pH 9.5 compared with the control (Fig. 3f). The water retention capacity of three willow cuttings' roots were in the order of *S. linearistipularis* > *S. gordejevii* > *S. matsudana*.

### Effects of alkaline stress on the chlorophyll content of three willow cuttings

As seen in Table 2, the changes in chlorophyll a, chlorophyll b and total chlorophyll content of the leaves of the three willow cuttings were consistent with a decreasing trend. *S. linearistipularis* showed the most significant decrease under alkaline stress at different pH values, while *S. matsudana* showed the smallest. At pH 8.0, the total chlorophyll contents of the three willow cuttings decreased by 1.40%, 10.30% and 17.70%, respectively, compared with the control.

**Table 2:** Effects of alkaline stress on chlorophyll a, chlorophyll b and total chlorophyll contents of three willow cuttings. (In statistical terms, the letters a, b, c, and d represent significant differences among the respective data at the *P ≤ 0.05* level).

Targets	Process	Tree species		
	pH	*Salix matsudana*	*Salix gordejevii*	*Salix linearistipularis*
Chlorophyll a content of leaves (mg/g)	7.2	1.390 ± 0.039a	1.428 ± 0.033a	1.617 ± 0.102a
	8.0	1.396 ± 0.169a	1.285 ± 0.133b	1.326 ± 0.268b
	8.5	1.258 ± 0.160b	1.179 ± 0.224b	1.184 ± 0.268b
	9.0	1.068 ± 0.193c	1.012 ± 0.242c	0.966 ± 0.260c
	9.5	0.907 ± 0.193d	0.934 ± 0.096c	0.893 ± 0.124c
Chlorophyll b content of leaves (mg/g)	7.2	0.540 ± 0.015a	0.547 ± 0.017a	0.579 ± 0.034a
	8.0	0.507 ± 0.074a	0.487 ± 0.057b	0.482 ± 0.083b
	8.5	0.451 ± 0.072b	0.447 ± 0.092b	0.454 ± 0.069b
	9.0	0.379 ± 0.069c	0.383 ± 0.090c	0.380 ± 0.091c
	9.5	0.334 ± 0.070d	0.351 ± 0.034c	0.350 ± 0.048c
Total chlorophyll content of leaves (mg/g)	7.2	1.930 ± 0.043a	1.975 ± 0.034a	2.197 ± 0.134a
	8.0	1.903 ± 0.239a	1.771 ± 0.186b	1.808 ± 0.348b
	8.5	1.709 ± 0.228b	1.625 ± 0.315b	1.637 ± 0.335b
	9.0	1.448 ± 0.261c	1.395 ± 0.331c	1.345 ± 0.346c
	9.5	1.241 ± 0.262d	1.284 ± 0.128c	1.243 ± 0.164c

### Effects of alkaline stress on photosynthetic gas exchange parameters in three willow cuttings

**Figure 4:**
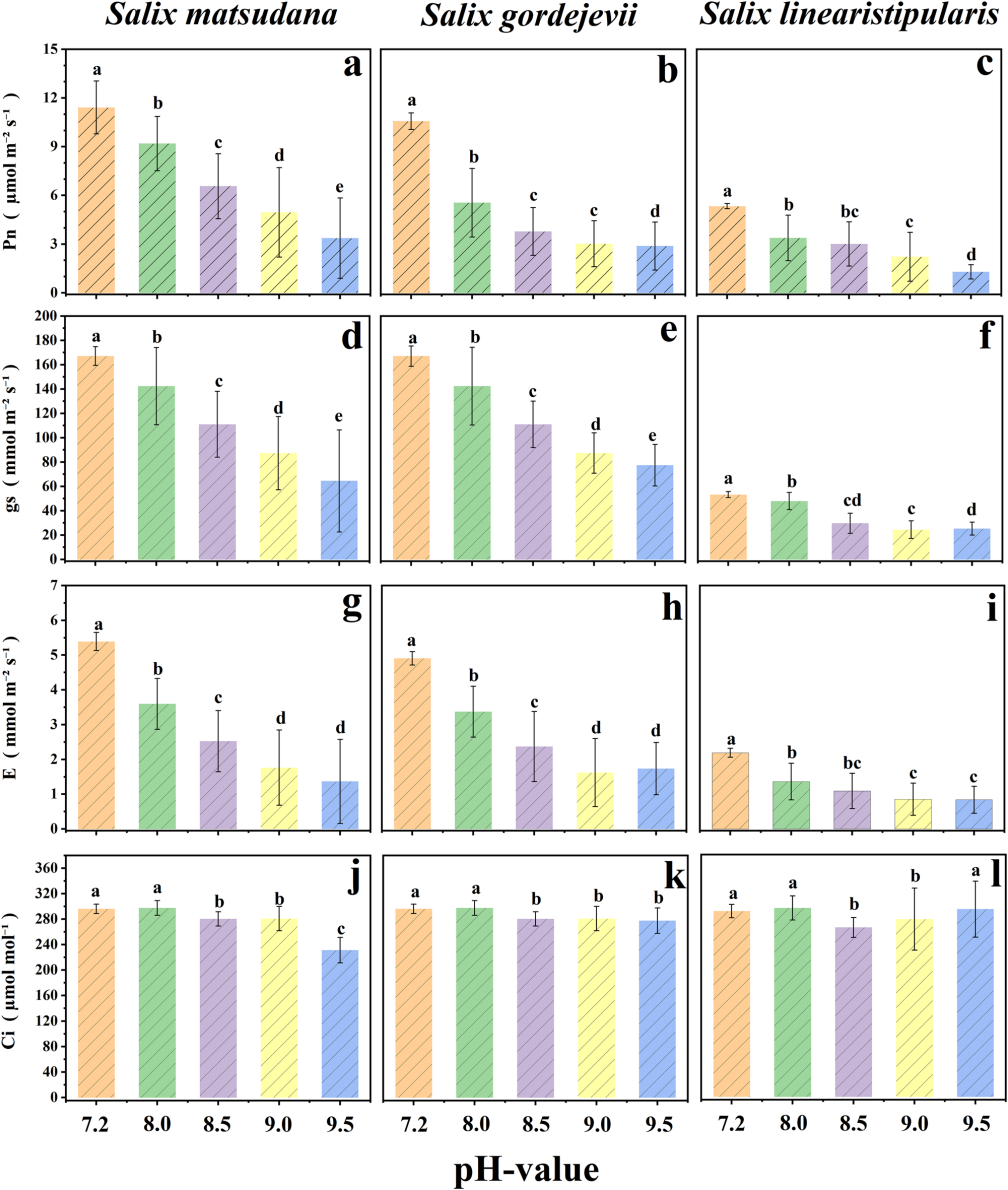
Effects of alkaline stress on net photosynthetic rate (**a**: *S. matsudana* ; **b**: *S. gordejevii*; **c**: *S. linearistipularis*), stomatal conductance (**d**: *S. matsudana*; **e**: *S. gordejevii*; **f**: *S. linearistipularis*), transpiration rate (**g**: *S. matsudana*; **h**: *S. gordejevii*; **i**: *S. linearistipularis*), and intercellular CO_2_ concentration (**j**: *S. matsudana*; **k**: *S. gordejevii*; **l**: *S. linearistipularis*) of three willow cuttings.

Photosynthesis is an essential pathway for biosynthesis and physiological metabolism during the growth and development of plant bodies and is more sensitive to changes in the external environment. Photosynthesis is an important indicator of the level of alkalinity resistance in plants. Figure 4 shows that *S. linearistipularis* had the lowest level of photosynthetic gas exchange among the three willow cuttings. The alkaline stress treatment concentration increased, and the leaf Pn, Gs and E of the three willow cuttings decreased significantly. Intercellular CO_2_ concentration of *S. matsudanau* leaves and *S. gordejevii* leaves showed a decreasing trend under alkali stress, and that of *S. linearistipularis* leaves showed a decreasing and then increasing trend (Fig. 4j, k, l).

Changes in the rate of decline of Pn in the leaves of the three willow cuttings differed with increasing pH. The rate of Pn decline in *S. gordejevii* and *S. linearistipularis* peaked between pH CK-8.0. They slowed down significantly between pH 8.0–9.5, whereas the rate of Pn decline in *S. matsudana* leaves was more stable throughout alkaline stress (Fig. 4a, b, c).

### Effects of alkaline stress on chlorophyll fluorescence kinetic parameters in three willow cuttings

#### Effects of alkaline stress quantum yield and energy partition ratio of three willow cuttings

**Figure 5:**
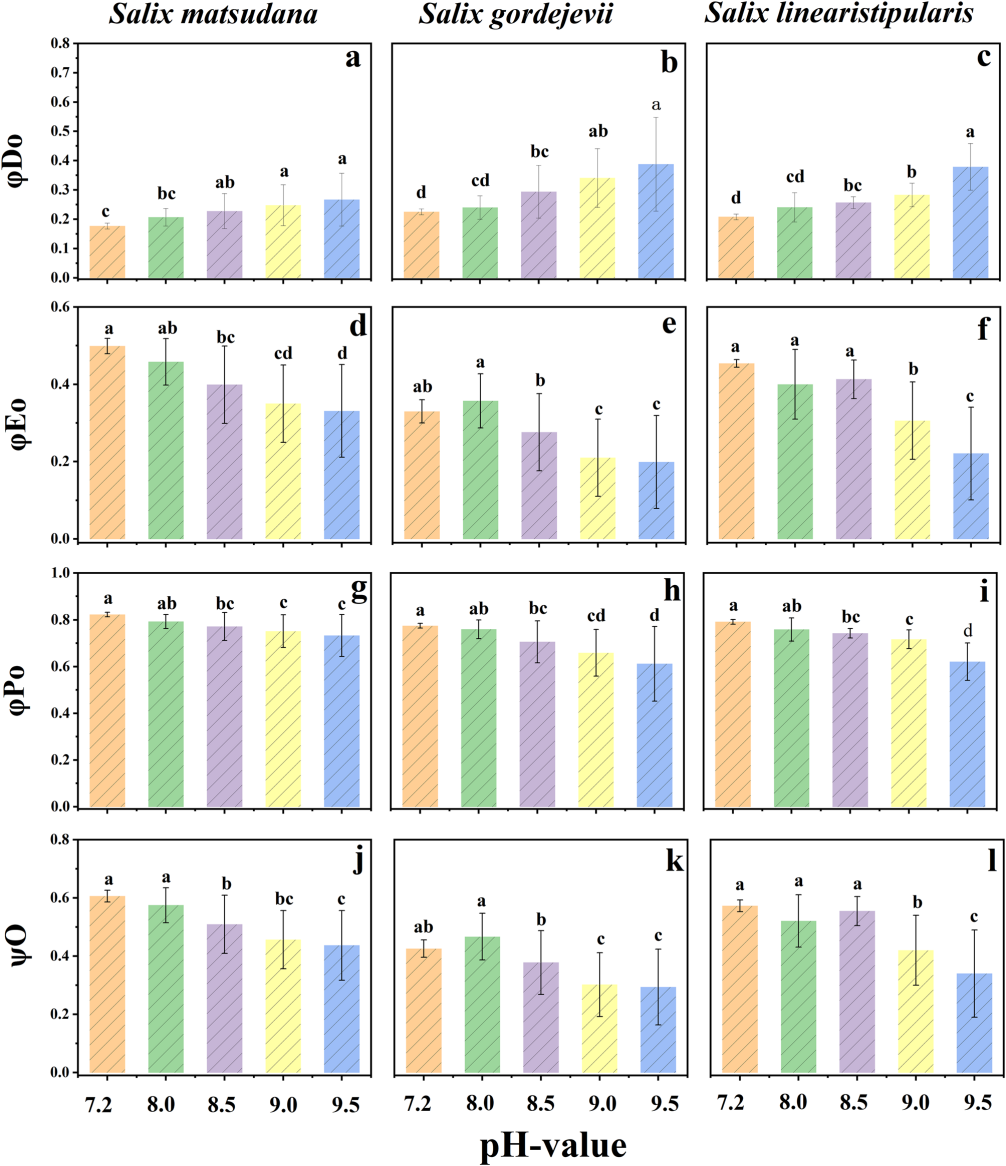
Effects of alkaline stress on the quantum ratio of heat dissipation φDo (**a**: *S. matsudana*; **b**: *S. gordejevii*; **c**: *S. linearistipularis*), the quantum efficiency of electron transfer from electron acceptors φEo (**d**: *S. matsudana*; **e**: *S. gordejevii*; **f**: *S. linearistipularis*), the maximal photochemical efficiency of PS II φPo (**g**: *S. matsudana*; **h**: *S. gordejevii*; **i**: *S. linearistipularis*), and the ratio of transferred electrons to trapped electrons φDo (**j**: *S. matsudana*; **k**: *S. gordejevii*; **l**: *S. linearistipularis*) of three willow cuttings.

As shown in Fig. 5, the trends of the quantum yield and energy allocation ratios of the leaves of the three willow cuttings under alkaline stress were generally consistent, all of which showed an overall gradual decrease in φPo, Ψo, and φEo and an overall gradual increase in φDo with the aggravation of the stress. There were differences in tolerance to alkaline stress among different species. *S. matsudana* showed better index stability under alkaline stress than the other two willows (Fig. 5a, d, g, j). Notably, *S. gordejevii* was able to better regulate its adaptive capacity to the adverse environment by changing the energy absorbed, converted, used for electron transfer and dissipated by heat radiation in the leaves at lower pH (pH < 9) under alkaline stress, during which its indices were less inhibited than those of the other two willows. From the point of view of energy utilisation, *S. gordejevii* had a more vital short-term alkaline stress tolerance ability. However, with further aggravation of the alkaline stress, the indices changed abruptly. The speed and magnitude of the changes were greater than those of *S. matsudana* and *S. linearistipularis*, and the degree of damage was significant (Fig. 5b, e, h, k).

#### Effects of alkaline stress on leaf performance indices and propulsion of three willow cuttings

As shown in Table 3, the changes in the PI_ABS_ and PI_CSm_ of *S. matsudana* leaves were the same, and both showed an overall decreasing trend with increasing pH. The DF_CSm_ of *S. matsudana* leaves showed an overall decreasing trend with increasing pH, and the differences were significant when the pH was 8.5, 9.0 and 9.5 compared with the control group. *S. gordejevii* leaf PI_ABS_, PI_CSm_ and DF_CSm_ all showed a trend of increasing and then decreasing with increasing pH. The three indices peaked when the pH was 8.0 and then decreased significantly with increasing pH in all treatments compared with the control group. The PI_ABS,_ PI_CSm_ and DF_CSm_ of *S. linearistipularis* leaves showed an overall decreasing trend with increasing pH, and each treatment group decreased significantly compared with the control group.

**Table 3:** Effects of alkaline stress on performance indices PI_ABS_, PI_CSm_ and driving force DF_CSm_ of three willow cuttings. (In statistical terms, the letters a, b, c, and d represent significant differences among the respective data at the *P ≤ 0.05* level).

Targets	Process	Tree species		
	pH	*Salix matsudana*	*Salix gordejevii*	*Salix linearistipularis*
PI_ABS_	7.2	4.690 ± 0.320a	1.141 ± 0.105a	2.622 ± 0.196a
	8.0	3.615 ± 1.645b	1.473 ± 0.689ab	2.156 ± 1.205a
	8.5	2.548 ± 1.555c	0.883 ± 0.644bc	1.535 ± 0.474b
	9.0	1.780 ± 1.780cd	0.519 ± 0.546cd	0.990 ± 0.606c
	9.5	1.592 ± 1.592d	0.472 ± 0.493d	0.465 ± 0.532c
PI_CSm_	7.2	105,800 ± 6360a	24,927 ± 1897a	48,269 ± 4624a
	8.0	80,698 ± 39699b	32,360 ± 16143ab	46,815 ± 27464a
	8.5	54,650 ± 34603c	19,444 ± 15121bc	29,496 ± 10632b
	9.0	37,571 ± 24906c	11,201 ± 12377c	18,883 ± 11604bc
	9.5	34,632 ± 21694c	10,729 ± 11688c	8444 ± 10231c
DF_CSm_	7.2	4.255 ± 0.044a	3.742 ± 0.055a	4.040 ± 0.071a
	8.0	4.114 ± 0.210ab	3.806 ± 0.229a	3.872 ± 0.341a
	8.5	3.912 ± 0.321bc	3.522 ± 0.412a	3.808 ± 0.197a
	9.0	3.755 ± 0.352c	3.203 ± 0.479c	3.576 ± 0.337b
	9.5	3.724 ± 0.400c	3.066 ± 0.724c	3.129 ± 0.500c

### Correlation analysis of indicators of three willow cuttings under alkaline stress

**Figure 6:**
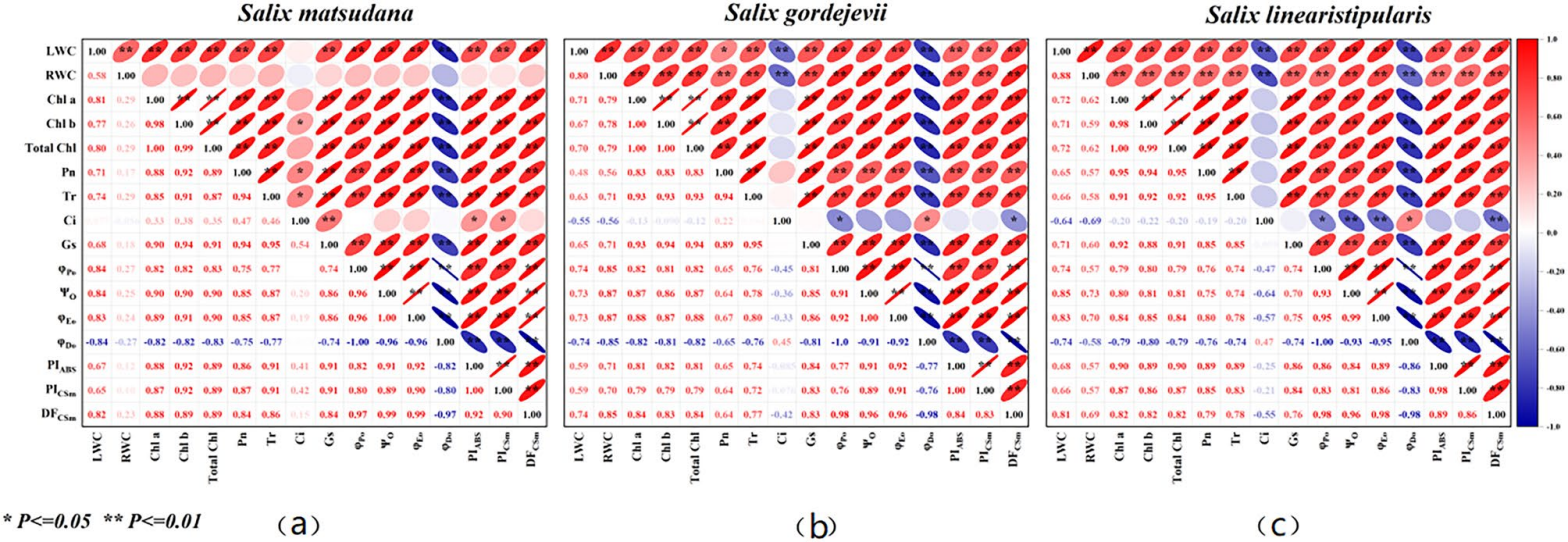
Correlation analysis of indicators of three willow cuttings under alkaline stress (**a**) *S. matsudana*; (**b**) *S. gordejevii*; (**c**) *S. linearistipularis.*

The relationship between 16 indicators of *S. matsudana* root and leaf water content, chlorophyll content, and photosynthesis can be determined by analysing a heatmap of the correlation matrix. From Fig. 6a, it is evident that in *S. matsudana,* there are 92 pairs of indicators with a highly significant correlation level $(P \leqq 0.01)$, 5 pairs with a significant correlation level $(P \leqq 0.05)$, and 22 pairs with no correlation or insignificant correlation. The correlation between root water content and other indicators was insignificant except for the highly significant correlation between root and leaf water content. The intercellular CO_2_ concentration in *S. matsudana* leaves had weak or no correlation with other indicators except for a highly significant correlation with stomatal conductance and significant correlations with chlorophyll b, net photosynthetic rate, transpiration rate, PI_ABS_ and PI_CSm_.

There were 105 pairs of highly significant correlations $(P \leqq 0.01)$, 4 pairs of significant correlations $(P \leqq 0.05)$, and 10 pairs of weak or nonexistent correlations among the indicators in *S. gordejevii* (Fig. 6). The intercellular CO_2_ concentration of *S. gordejevii* showed weak or no correlation with most indicators, except for a negative correlation with root and leaf water content and significant correlations with φPo, φDo, and DF_CSm_. The correlation between Pn and leaf water content did not reach the level of significance, which was different from that of *S. matsudana* and *S. linearistipularis*.

In *S. linearistipularis*, 109 pairs of indicators had highly significant correlations $(P \leqq 0.01)$, two pairs of indicators had significant correlations $(P \leqq 0.05)$, and the correlations between the other indicators were weak or nonexistent (Fig. 6c). The cellular CO_2_ concentration of *S. linearistipularis* leaves showed a highly significant negative correlation with root and leaf water content, Ψo, φEo and DF_CSm_, a significant negative correlation with φPo, a significant positive correlation with φDo, and a weak or nonexistent correlation with the other indicators. Except for the intercellular CO_2_ concentration, there were highly significant correlations between the other indicators.

## Discussion

### Analysis of changes in root and leaf dry weight of three willow cuttings under alkaline stress

Often forest trees trade reduced growth for survival in alkaline environments. The change in biomass is an integrated expression of the plant's response to stress and is one of the direct indicators representing the plant's alkaline tolerance. As one of the main organs for material exchange in plants, the growth of the root system is closely related to the growth and development of the above-ground parts, whether the root system can function properly, and the efficiency of water and nutrient utilisation by plants^15,16^. However, because of its direct contact with adversity stress, it can be the earliest to feel the signals of adversity stress and become the first and foremost site of damage^17^. It has been shown that the metabolic regulation of roots is the first barrier for plants to cope with saline and alkaline stress^18,19^, and in particular, the alkaline resistance of plants mainly depends on roots^20^. This study found that the dry weights of roots and leaves of three willow cuttings generally tended to decrease during alkaline stress. The dry weight of the root system of *S. matsudana*, which is highly alkaline tolerant was always maintained at a high level. *S. gordejevii* root dry weight also maintained a stable state at higher levels, although it changed significantly under low pH stress. *S. linearistipularis* root system dry weight remained low and decreased significantly at pH 8.5. Therefore, the alkaline tolerance of the three willow cuttings was thus *S. matsudana* > *S. gordejevii* > *S. linearistipularis*.

### Analysis of changes in root and leaf water content of three willow cuttings under alkaline stress

Salinity stress can lead to osmotic pressure imbalance in the plant, preventing water uptake and, in severe cases, leading to water exudation or even plant death^21^. In this experiment, the water content of the root systems of *S. gordejevii* and *S. linearistipularis* decreased by approximately the same amount. In contrast, the water content of the root system of *S. matsudana* decreased by the most significant amount. However, the water content of *S. matsudana* leaves was able to remain stable. *S. matsudana* reduced root water uptake as much as possible without affecting the growth of the root system, which is an essential mechanism for plant adaptation to saline and alkaline stress. This supports Jia's study on the impact of alkaline stress on hickory's photosynthetic properties^22^. Yang et al. pointed out in their study on alfalfa that plant water loss can be used as a rapid and economical osmotic adjustment to cope with osmotic imbalance brought about by saline and alkaline stress^23^. The results of this study showed that the stability of the leaf water content of the three willow cuttings was a vital reflection of the strength of their resistance. *S. matsudana* leaf water content remained stable throughout the alkaline stress period, *S. gordejevii* leaves retained water better under low-concentration stress, and *S. linearistipularis* leaves retained water the least. This difference in water uptake among willow species may be due to variations in their ability to accumulate osmoregulatory substances, which affect osmotic pressure.

### Analysis of changes in photosynthetic pigments and the photosynthetic system of three willow cuttings under alkaline stress

Photosynthesis in plant leaves depends mainly on the absorption, transfer and conversion of light energy in Thylakoid Membrane^24^. Studies have shown that photosystem II (PSII) is the most sensitive to salinity stress, and chloroplasts are one of the most essential organelles in plant PS II that respond to salinity stress^25^. Chlorophyll a and chlorophyll b are concentrating pigments that are not photochemically active. They are mainly responsible for absorbing light energy and transferring it to the unique chlorophyll pair in the reflection centre during photosynthesis^26^. Changes in the external environment will directly affect the chlorophyll content of leaves, which in turn will impact the photosynthetic function of plants. Therefore, chlorophyll content has become an important reference index for plant salinity tolerance^27^. In addition, Mg^2+^ and Fe^2+^, as essential elements for chlorophyll synthesis and chloroplast structure, precipitate at high pH, which is one of the reasons for the decrease in plant chlorophyll content due to alkaline stress^28^. In this study, we found that during alkaline stress, the photosynthetic pigment content of the three willow species varied in a gradual decline with increasing pH during alkaline stress. *S. matsudana* was able to ensure the relative stability of its photosynthetic pigment content during alkaline stress and was less affected by alkaline stress, followed by *S. gordejevii*. Furthermore, *S. linearistipularis* showed the most significant decrease in photosynthetic pigment content. The total chlorophyll content of the three willow cuttings showed significant differences in their sensitivity to low concentrations of alkaline stress, with *S. linearistipularis* > *S. gordejevii* > *S. matsudana*. This indicates that alkaline stress destroys the plant environment, affects the physiological functions of the plant, reduces the stability of the cyst-like membrane, significantly inhibits the synthesis of chlorophyll, and accelerates the decomposition of chlorophyll by increasing chlorophyll hydrolase, thus weakening the absorption of light energy by chlorophyll^29^. However, there were significant differences in the magnitude and rate of decline in chlorophyll content of the three willow cuttings under alkaline stress, which may be related to the resistance of the tree species to saline and alkaline stress.

Photosynthesis, as a critical anabolic process of plant material energy sources, not only provides energy for the average growth, development and physiological metabolism of plants but is also more sensitive to adverse environments and is one of the critical indicators for determining the damage status of plants^30^. Alkaline stress is one of the critical factors for the inhibition of plant photosynthesis compared to salt stress. In addition to ionic toxicity and osmotic stress from high concentrations of salt ions in the plant body, high pH stress in the interroot is also one of the critical factors for inhibiting plant photosynthesis^31^. Studies have demonstrated that plants can recover from the effects of salt stress. The degree of recovery from damage to the acceptor side of the PS II reaction centre under salt stress was close to 100%. The degree of recovery from damage to the donor side was less than 85%.

In contrast, the effects of alkaline stress on the photosynthetic system of phytomass were not reversible, which may be related to the effect of high pH of alkaline stress on the plant^32^. In addition, alkaline stress inhibits chlorogenesis and reduces the photosynthetic area and carbon assimilation in monocots, which results in physiological metabolic disorders and the accumulation of toxic substances^33^. Salinity stress inhibits energy-related processes, such as photosynthesis, carbohydrate metabolism, and the TCA cycle^34^.

The net photosynthetic rate, as a critical indicator of photosynthesis, can straightforwardly reflect the material production capacity of plants per unit leaf area^35^. Based on previous studies, limiting factors affecting photosynthesis under adverse stress can be categorised into two main groups: stomatal limitation and nonstomatal limitation^36^. In this study, the leaf Pn, Gs and E of all three willow cuttings decreased significantly with increasing pH. Among them, *S. matsudana* was better able to maintain the stability of its own Pn under more substantial stress concentrations. Furthermore, *S. gordejevii* and *S. linearistipularis* were significantly inhibited at the beginning of alkaline stress. The reduction in transpiration rate and stomatal conductance may be an adaptive response to the reduction in water content, which is consistent with the reduction in willow leaf water content in the results of this experiment^37^. Reducing the transpiration rate as much as possible without affecting the photosynthetic rate is essential for plant adaptation to adversity^38^. Leaf Ci decreased with decreasing Gs at lower pH in three willow cuttings, showing typical stomatal factor limitation^39^. At higher pH for the three willow cuttings, *S. matsudana* and *S. gordejevii* leaf Ci did not change significantly with decreasing Gs. *S. linearistipularis* leaf Ci increased with decreasing Gs, showing typical nonstomatal factor limitation. The changes in Ci of *S. matsudana* and *S. gordejevii* leaves indicated that the two types of willows were less affected by nonstomatal limiting factors under high alkaline stress, which differed from the results of previous studies and might be highly related to the unique salinity resistance mechanism of the two types of willows.

### Analysis of changes in chlorophyll fluorescence kinetic parameters in three willow cuttings under alkaline stress

φPo, Ψo, φEo and φDo reflect the energy allocation ratio of plants. In this study, at lower pH at the beginning of alkaline stress, all fluorescence indices of the three willow cuttings showed no significant difference compared with the control group, indicating that the low concentration of alkaline stress had less effect on the energy allocation in the PS II reaction centre of the three willow cuttings, which was the same as the results of Zhang et al.^40^. With stress intensification, φPo, Ψo, and φEo significantly decreased while φDo significantly increased in *S. gordejevii* and *S. linearistipularis*, and the magnitude of changes was positively correlated with stress intensity. *S. matsudana* φPo, Ψo, φEo and φDo had the same trend of change as those of the other two willows, but the magnitude of change was smaller, and φPo differed from the control only at pH 9.5. The three willow cuttings adjusted the energy allocation ratios of their own PS II reaction centres under alkaline stress, i.e., the quantum ratio of energy absorbed by antenna pigment cells for heat dissipation increased and the energy share of photochemical reactions decreased^41^. Plants rely on light energy through photosynthesis to synthesise organic matter. However, too much light energy can harm their photosynthetic apparatus. To prevent damage, excess energy is dissipated through the lutein cycle for photoprotection, according to Barbara et al.^42^. To reduce the damage to the photosynthetic apparatus caused by excess light energy, plants will also appropriately reduce chlorophyll content to reduce the capture of excess light energy without affecting photosynthesis, which is also a self-regulatory mechanism for plants to cope with stress^43,44^. In conclusion, the three willow cuttings showed different degrees of decreases in φPo, Ψo and φEo, indicating that the photosynthetic apparatus was significantly damaged and that the ability of the PSII receptor side Q_A_ to transfer electrons and the ability of Q_B_ and PQ to be reduced were reduced, as evidenced by the reduced opening of the active reaction centres and the inhibition of the electron transfer process^45^. Compared with the three willow cuttings, *S. matsudana* had the most vital ability to maintain its stability under alkaline stress; *S. gordejevii* was more tolerant at lower alkaline stress concentrations, but the degree of damage increased rapidly with a further increase in alkaline stress; and *S. linearistipularis* was the least resistant throughout the alkaline stress process.

Plants are prone to photoinhibition or exacerbation in adverse environments, and PI_ABS_ can comprehensively reflect the activity of photosystems through the three aspects of absorption, capture and electron transfer of light energy under adverse conditions^46^. In this study, the overall trend of decreasing PI_ABS_, PI_CSm_ and DF_CSm_ in the leaves of the three willow cuttings showed a more obvious decreasing trend with the increasing degree of alkaline stress, among which *S. matsudana* showed a minor change, *S. gordejevii* showed a more vital ability to tolerate the low concentration of stress, and *S. linearistipularis* showed the most apparent decreasing trend. This indicates that alkaline stress seriously affects the absorption and utilisation of light energy and leads to a decrease in the basal driving force, and the degree of reduction in PSII activity varies among different species of willow leaves. The stress triggered photoinhibition, reversible inactivation or irreversible degradation of the PSII reaction centre, reduced light energy conversion efficiency and impaired functioning of the photosynthetic apparatus in willow leaves, thus limiting the normal conduct of photosynthesis.

### Analysis of correlation differences among indicators of three willow cuttings under alkaline stress

Correlation analysis among indicators of three willow cuttings under alkaline stress. This study showed no significant correlation between the root water content of *S. matsudana* and other indicators, except for a significant positive correlation with leaf water content $(P \leqq 0.05)$. Unlike *S. matsudana,* the correlation between root system water content and other indicators of *S. gordejevii* and *S. linearistipularis* reached a significant level $(P \leqq 0.01)$. The difference in correlations among the three willow cuttings may be related to the existence of unique salinity resistance mechanisms in *S. matsudana*. Compared with *S. gordejevii* and *S. linearistipularis*, *S. matsudana* has better drought resistance, which reduces the effect of alkaline stress on the plant by reducing the water content of the root system and synthesising more osmoregulatory substances to maintain the water content of the leaves without affecting the normal physiological metabolism of the plant. Some studies have shown that the decrease in the water content of alfalfa cuttings under alkaline stress is not only a result of osmotic stress but also may be due to the damage of high pH to the root system structure of the plant body and osmotic regulatory substances^23^. Suitable water content may enable salt-resistant plants to develop less energy-consuming osmotic pressure, thus increasing the resistance of saline plants to stress^47^.

It was also observed in this study that the intercellular CO_2_ concentration in *S. matsudana* was significantly and positively correlated with the net photosynthetic rate $(P \leqq 0.05)$, transpiration rate $(P \leqq 0.05)$, and stomatal conductance $(P \leqq 0.01)$ and was not correlated with root and leaf water contents. In contrast, the intercellular CO_2_ concentrations in *S. gordejevii* and *S. linearistipularis* were not correlated with the net photosynthetic rate, transpiration rate, and stomatal conductance, root and leaf water content was significantly negatively correlated $(P \leqq 0.01)$. The results indicated that stomatal factors were the main limiting factors for the growth of *S. matsudana* under alkaline stress. In contrast, nonstomatal factors were the main limiting factors for the growth of *S. gordejevii* and *S. linearistipularis* under alkaline stress.

## Conclusion

Alkaline stress affected root and leaf dry weight, root and leaf water content, chlorophyll content, photosynthesis and chlorophyll fluorescence kinetic parameters in all three willow cuttings. To some extent, the three willow cuttings can cope with the damage caused by alkaline stress by appropriately reducing the root water content while increasing the water-holding capacity of the leaves. Stronger alkaline stress also inhibited chlorophyll synthesis and accelerated chlorophyll decomposition. At low concentrations of alkaline stress, stomatal closure was the leading cause of decline in all three willow cuttings. In contrast, at high concentrations of alkaline stress, nonstomatal factors were the main factors limiting photosynthesis in all three willow cuttings. *S. matsudana* and *S. linearistipularis* photosynthesis were less affected by nonstomatal limiting factors, while *S. linearistipularis* photosynthesis was significantly nonstomatal limited. In terms of chlorophyll fluorescence kinetic parameters, alkaline stress degraded or inactivated the leaf PSII reaction centre and caused damage to both the donor and acceptor sides of PSII, triggering a series of responses, such as photoinhibition, reduction in light energy conversion efficiency and impaired functioning of the photosynthetic apparatus in the leaves of the three willow cuttings. The correlation between the root water content of *S. matsudana* and the indicators once again illustrated the idea that plants can cope with alkaline stress by reducing the root water content. Furthermore, a comparison of the correlations between Ci and Pn, Gs and E showed that nonstomatal limiting factors had less inhibitory effects on the growth of *S. matsudana* and that the growth of *S. gordejevii* and *S. linearistipularis* was significantly nonstomatal-limited.

The adaptive mechanisms of the three willow cuttings to alkaline stress differed. The performance of the three willow cuttings showed that *S. matsudana* could better maintain the relative stability of the indicators at higher alkaline stress concentrations, followed by *S. gordejevii,* which showed a stronger ability to tolerate low alkaline stress concentrations. However, the rate of change in the indicators increased rapidly with further stress intensification. *S. linearistipularis* was the least adaptive to the regulation of alkaline stress.

## Data Availability

Data will be made available on request. You can send us an email to get the raw data. E-mail address: adwancer10086@126.com.
